# Reproducible and opposing gut microbiome signatures distinguish autoimmune diseases and cancers: a systematic review and meta-analysis

**DOI:** 10.1186/s40168-022-01373-1

**Published:** 2022-12-09

**Authors:** Md Zohorul Islam, Melissa Tran, Tao Xu, Braden T. Tierney, Chirag Patel, Aleksandar David Kostic

**Affiliations:** 1 Section on Pathophysiology and Molecular Pharmacology, Joslin Diabetes Center, Boston, MA USA; 2 Department of Microbiology, Harvard Medical School, Boston, MA USA; 3 Section of Experimental Animal Models, Department of Veterinary and Animal Sciences, University of Copenhagen, Copenhagen, Denmark; 4 Department of Biomedical Informatics, Harvard Medical School, Boston, MA USA; 5 Section on Islet Cell and Regenerative Biology, Joslin Diabetes Center, Boston, MA USA

**Keywords:** Cancer, Autoimmunity, Microbiome, Metagenome, Health

## Abstract

**Background:**

The gut microbiome promotes specific immune responses, and in turn, the immune system has a hand in shaping the microbiome. Cancer and autoimmune diseases are two major disease families that result from the contrasting manifestations of immune dysfunction. We hypothesized that the opposing immunological profiles between cancer and autoimmunity yield analogously inverted gut microbiome signatures. To test this, we conducted a systematic review and meta-analysis on gut microbiome signatures and their directionality in cancers and autoimmune conditions.

**Methodology:**

We searched PubMed, Web of Science, and Embase to identify relevant articles to be included in this study. The study was conducted in accordance with the Preferred Reporting Items for Systematic Reviews and Meta-Analyses (PRISMA) statements and PRISMA 2009 checklist. Study estimates were pooled by a generic inverse variance random-effects meta-analysis model. The relative abundance of microbiome features was converted to log fold change, and the standard error was calculated from the *p*-values, sample size, and fold change.

**Results:**

We screened 3874 potentially relevant publications. A total of 82 eligible studies comprising 37 autoimmune and 45 cancer studies with 4208 healthy human controls and 5957 disease cases from 27 countries were included in this study. We identified a set of microbiome features that show consistent, opposite directionality between cancers and autoimmune diseases in multiple studies. *Fusobacterium* and *Peptostreptococcus* were the most consistently increased genera among the cancer cases which were found to be associated in a remarkable 13 (+0.5 log fold change in 5 studies) and 11 studies (+3.6 log fold change in 5 studies), respectively. Conversely, *Bacteroides* was the most prominent genus, which was found to be increased in 12 autoimmune studies (+0.2 log fold change in 6 studies) and decreased in six cancer studies (−0.3 log fold change in 4 studies). Sulfur-metabolism pathways were found to be the most frequent pathways among the member of cancer-increased genus and species.

**Conclusions:**

The surprising reproducibility of these associations across studies and geographies suggests a shared underlying mechanism shaping the microbiome across cancers and autoimmune diseases.

Video Abstract

**Supplementary Information:**

The online version contains supplementary material available at 10.1186/s40168-022-01373-1.

## Introduction

Development of cancer and autoimmune diseases is influenced by contrasting manifestations of immune dysfunction. Immunosubversion is the prime immune functional defect in cancer pathogenesis [1], while hyperactivation of the immune system against self-antigens is the main pathogenic alteration in autoimmune pathogenesis [2]. This contrasting immune landscape between autoimmunity and cancer is mainly explained by immunological tolerance [3]. Autoreactive immune cells escaping from the central tolerance mechanism and impaired peripheral tolerance are the hallmarks of autoimmunity. In contrast, the immune system’s weakness to cancer’s immune evasion and induction of peripheral tolerance are considered hallmarks of cancer [4]. Immune cells, regulatory factors, and cytokines modulate the development and progression of cancers and autoimmunity through immune homeostasis and peripheral tolerance. T regulatory cells (Tregs) are the major immune cells found with increased frequency in the tumor microenvironment and are believed to be negatively associated with antitumor immune responses, promotion of malignancy, and a worsening prognosis in many types of cancers [5–7]. Conversely, Treg frequency has been found to be decreased in diverse types of autoimmune diseases such as type 1 diabetes (T1D) [8], arthritis [9], and systemic lupus erythematosus [10–12], which explains the loss of peripheral tolerance in autoimmune pathogenesis [13]. Another critical regulatory component is the immune checkpoints, such as CTLA-4 and PD-1, which are less expressed by immune cells in autoimmunity and highly expressed by immune cells and tumor cells in cancers [14–16]. For instance, CTLA-4 deficiency is associated with immune dysregulation and aberrant autoimmunity [17, 18]. On the other hand, blocking the CTLA-4 by Anti-CTLA-4 antibodies was shown to remove the immune barrier to cancer and demonstrated as promising cancer therapy [19]. Furthermore, many proinflammatory and immunosuppressive cytokines are found to be increased or decreased in an opposite manner between cancer and autoimmunity, such as IL-10 and TGF-beta [3]. However, the presence of opposite immune landscape between autoimmunity and cancer is not always constant. There are some aspects where the immune responses are actually quite similar in cancer and autoimmunity. For instance, a proinflammatory type of immune landscape are observed in the early stage of colorectal [20] and other cancers [21]. Although genetic background is one of the major predisposing factors both in cancer and autoimmunity, other environmental factors, such as the gut microbiome, have recently been found to be strongly associated with both cancers and autoimmune diseases [22, 23]. The gut microbiome contributes to the host’s “immune education” and lymphoid tissue development [24, 25] and is hypothesized to be capable of promoting the immune system, as seen in autoimmune diseases or cancers.

We hypothesized that the gut microbiome would mirror the inverse immunologic relationship between autoimmune disease and cancer. In other words, certain gut microbiome features would be shared across autoimmune diseases and others shared across cancers, with those shared by both conditions demonstrating opposite-sign associations (e.g., depleted in cancer and enriched in autoimmune disease). Although many independent studies have surveyed microbiome associations with autoimmune diseases and cancer, there is a lack of large-scale, evidence-based, systematic analysis of research findings on this topic. The observation of consistent microbiome associations across multiple studies and in diverse geographical locations is paramount to painting the landscape of robust and reproducible microbiome associations in human disease, and comparing associations across diseases is likely to yield novel insights into the underlying and differentiating biology of the phenotypes in question.

We identified 82 published microbiome studies and synthesized the results of the microbiome features that are associated with multiple cancer and autoimmune diseases. Furthermore, we explored the functional potential of the identified gut microbiome features in association with cancer and autoimmunity. These findings revealed a novel set of gut microbiome signatures showing a robust association with multiple cancer or autoimmune disease conditions across multiple studies, which may serve as hypotheses for future studies investigating the relationship between the microbiome and immunological state.

## Methods

### Literature source and search strategy

A systematic database search strategy was employed to identify microbiome studies in cancer and autoimmune diseases. On March 26, 2020, three electronic databases (PubMed, Web of Science, and EMBASE) were searched for relevant publications until the date of the search. The first author (MZI) searched the database. The database search was conducted separately for autoimmune diseases and cancers. The microbiome and disease-related search terms were joined together using the Boolean operator “AND” and “OR.” Medical Subject Headings (MeSH) terms were used for PubMed, and a combination of general search terms was used for the Web of Science and Embase databases. A detailed description of the database search terms is listed in Supplementary Table 1.

### Study eligibility criteria

Studies published in English from 1 January 2008 to 26 March 2020 were considered. The major inclusion criteria included the following: (1) the study was on the association between the gut microbiome with either cancer or autoimmune diseases (i.e., colorectal cancer and adenoma, gastric cancer and adenocarcinoma, pancreatic cancer and ductal adenocarcinoma, prostate cancer, cervical cancer, lung cancer, thyroid papillary cancer, breast cancer, hepatocellular carcinoma, gastrointestinal tract (GIT) neoplasia, type 1 diabetes, autoimmune arthritis, multiple sclerosis, systemic lupus erythematosus, Graves’ disease, primary Sjögren’s syndrome, pemphigus vulgaris, anti-N-methyl-D-aspartate receptor (NMDAR) encephalitis, and autoimmune hepatitis), (2) the study subjects were human, (3) the study had clear demarcation of healthy control and disease cases, and (4) the DNA sequencing method was 16s rRNA or shotgun. Cross-sectional, case-control, or longitudinal cohort studies were considered to be eligible if they had samples from healthy control and disease cases. Studies were excluded if they had insufficient taxonomic classification below genus, lack statistical comparison of microbiome taxonomic abundance between cases and controls, and contained cases that underwent treatment with antibiotics or failed to meet the inclusion criteria mentioned above. We did not include conference abstracts, reports, and experimental and intervention studies.

### Study screening and selection

All publications identified by the systematic database search were first screened for duplicates. After duplicate removal, the titles and abstracts of all publications were independently screened by two authors (MZI and MT) to identify potential studies for full-text screening.

### Data extraction

We developed and evaluated a pilot data extraction spreadsheet before the final data extraction. Full-text articles were screened for data extraction by one author (either MZI or MT). Independent overlapping data extraction was performed for 5% of the studies. Any disagreements between the data extractors were resolved by consensus. Data were extracted on the first author, year of publication, country, type of disease, method of disease diagnosis, specimen types, number of cases and controls, age of the subject, characteristics of control, DNA sequencing type and platform, availability and source of sequencing data, alpha diversity, statistically significant genus and species and their corresponding *p*-values, the relative abundance of genus and species, and statistically significant metabolic pathways and their corresponding *p*-values.

### Maintenance of study standard and quality assessment of individual study

We conducted this systematic review in accordance with Preferred Reporting Items for Systematic Reviews and Meta-Analyses (PRISMA) statements [26] and PRISMA 2009 checklist (Supplementary Table 2). We did not publish a protocol for this systematic review and meta-analysis. All included studies were independently assessed for quality and bias using the Newcastle-Ottawa Scale (NOS) [27]. The risk of bias in each study was assessed in three major domains: study selections, compatibility, and ascertainment of exposure.

### Data analysis

To identify shared and unique microbiome features (genus, species, and metabolic pathways) between autoimmune and cancer studies, we extracted genus, species, and predicted metabolic pathway data that were already analyzed in published studies. We considered all the individual studies as an independent observation and extracted the significant (false discovery rate, FDR adjusted *p*-value < 0.05 or nominal *p*-value < 0.05 where FDR adjusted *p*-value was absent) associated microbiome features found between cases and controls. This approach enabled us to exclude spurious microbiome features and to aggregate the most meaningful features from multiple studies. We first removed microbiome features that were found only once in the 82 included studies. We then defined whether a feature is “increased” or “decreased” among the cases of cancer and autoimmune studies. We defined a microbiome feature as “increased in cancer” if it was found to be significantly increased among the cancer cases in more than one cancer study and in at least 70% of the cancer studies that identify the feature as significant. On the contrary, we considered a microbiome feature as “decreased in cancer” if it was decreased among the cancer cases in more than one cancer study and in at least 70% of the cancer studies that identify the feature as significant. In the same way, we defined the microbiome features as “increased in autoimmune” and “decreased in autoimmune.”

Data were synthesized and summarized in Microsoft Office Excel. Meta-analyses on the relative abundance of the bacterial genus were conducted in R version 4.0.2 [28] using R package metafor version 2.4-0 [29] and meta version 4.13-0 [30]. The relative abundance data were converted to log fold-change, and the standard error was calculated from the *p*-values, sample size, and log fold change using R package dmetar [31]. The standard error was calculated using “se.from.p” function [se.from.p(effect.size = log fold-change, *p* = pvalue, *N* = sample size, effect.size.type = “ratio”). Generic inverse variance random-effect meta-analysis was performed using “metagen” function [*metagen(Log fold-change, standard error of fold-change, studlab, data = data, subset = genus == "genus name," byvar = disease type, comb.fixed = FALSE, comb.random = TRUE, hakn = FALSE, prediction = FALSE, print.byvar = FALSE, sm=""*]. The DerSimonian-Laird random-effects method was used to combine the study estimates [32]. The forest plots were generated using the “forest” function in R metafor package [*forest(Random effect model, …*]. The full meta-analysis pipeline is available in a GitHub repository (https://github.com/mdzohorulislam/Cancer-autoimmunity_meta-analysis).

## Results

### Results of electronic database search and study selection

**Fig. 1:**
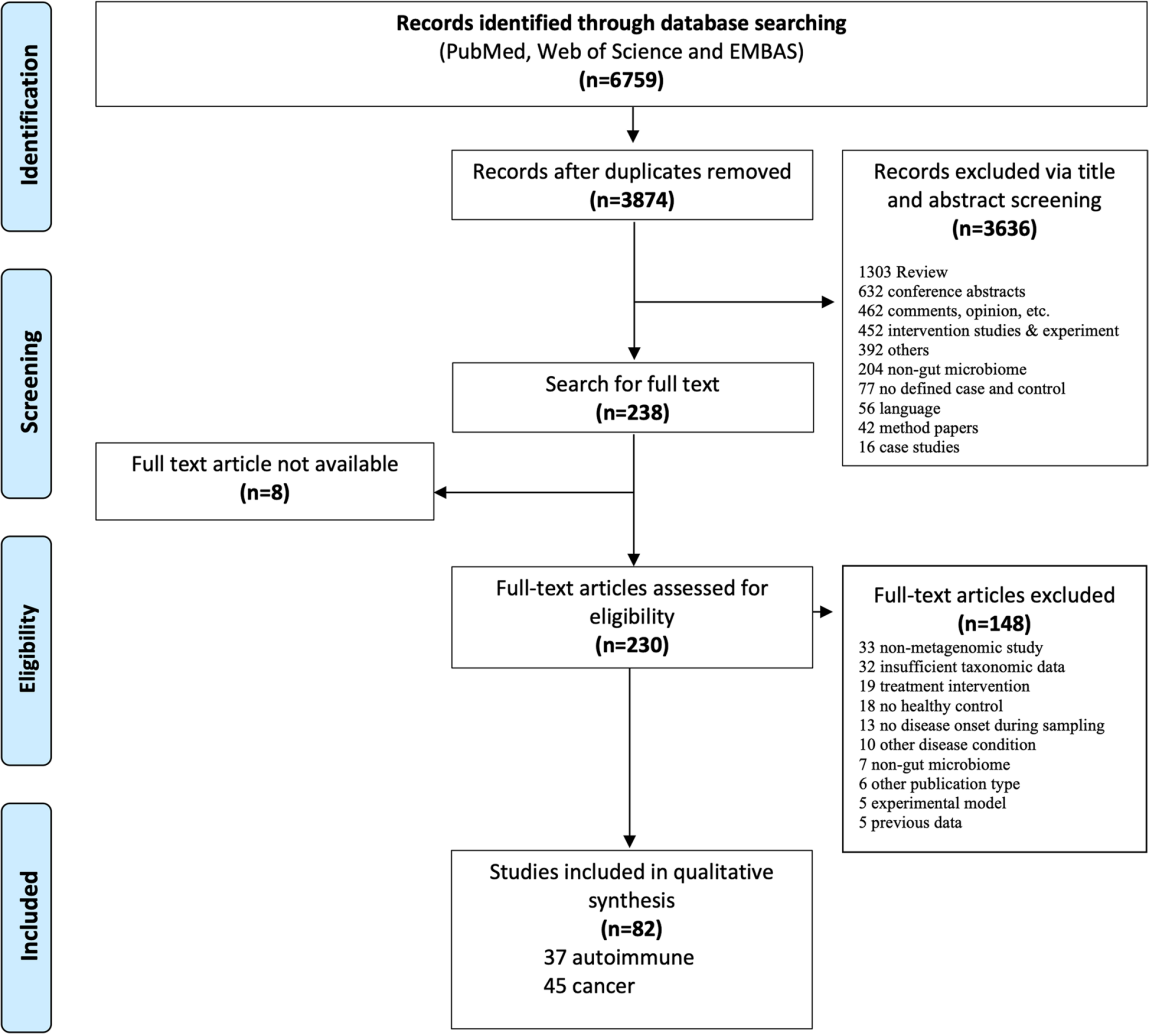
PRISMA flow diagram of study selection

We identified 3874 potentially relevant publications by searching three electronic databases (Fig. 1, “Methods”). We screened the title and abstract of all publications identified in the initial database search. Primary screening identified 238 studies for full-text retrieval and eligibility checking. A total of 82 case-control studies were included (Supplementary Tables 3–4).

### Characteristics and quality assessment of the included studies

**Fig. 2:**
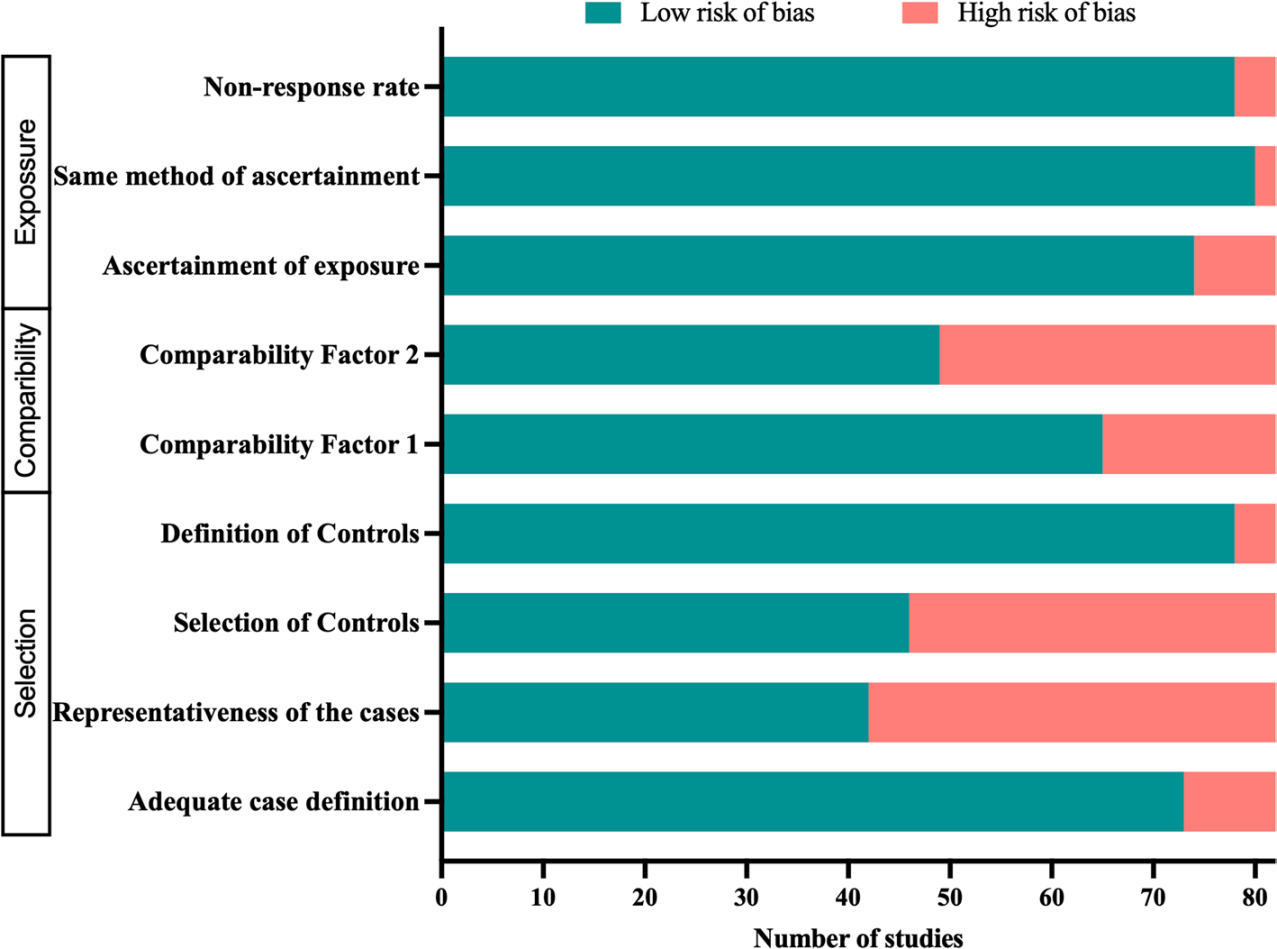
Summary of the results of risk-of-bias assessment by the Newcastle-Ottawa Scale (NOS)

We included 37 autoimmune and 45 cancer studies, amassing 4208 healthy human controls and 5957 disease cases from 27 countries (Table 1, Supplementary file 1). We captured nine types of autoimmune diseases: type 1 diabetes (*n* = 13) [33–45], arthritis (*n* = 9) [46–54], multiple sclerosis (*n* = 7) [46, 55–60], systemic lupus erythematosus (*n* = 4) [61–64], Graves’ disease (*n* = 2) [65, 66], primary Sjögren’s syndrome (*n* = 1) [63], pemphigus vulgaris (*n* = 1) [67], anti-NMDAR encephalitis (*n* = 1) [68], and autoimmune hepatitis (*n* = 1) [69]. The cancer studies constituted ten types of cancer including colorectal cancer and adenoma (*n* = 27) [70–96], gastric cancer and adenocarcinoma (*n* = 6) [97–102], pancreatic cancer and ductal adenocarcinoma (*n* = 3) [103–105], prostate cancer (*n* = 2) [106, 107], cervical cancer (*n* = 2) [108, 109], lung cancer (*n* = 1) [110], thyroid papillary cancer (*n* = 1) [111], breast cancer (*n* = 1) [112], hepatocellular carcinoma (*n* = 1) [113], and GIT neoplasia (*n* = 1) [114]. Overall, the majority of the studies (79%) have low risk of bias (based on the nine criteria of the Newcastle-Ottawa Scale) (Fig. 2). However, there was a high risk of bias in some indicators such as bias due to lack of representative cases (49%) and improper selection of controls (44%). The lack of representative case bias was mainly due to the absence of consecutive or obviously representative series of cases, and the improper selection of controls bias was due to inclusion of controls from hospital or no description about the selection of study control.

**Table 1:** Summary of study characteristics

Categories	Disease	Country (ISO2)	No. of study	No. of case	No. of control	Age-range (years)	Sequencing type (no. of study)
Autoimmune	Type 1 diabetes	AU, AZ, CN, FI, DE, JO, MX, NG, PL, ES, SD, SE, NL, UK, US	13	568	893	1–47	16s (12) and shotgun (1)
	Arthritis^a^	CA, CN, FI, FR, IN, IT, US	9	438	339	5–71	16s (8) and shotgun (1)
	Multiple sclerosis	CN, BE, JP, US	7	272	328	12–50	16s
	Systemic lupus erythematosus	CN, ES, NL	4	156	253	45–50	16s
	Graves’ disease	CN	2	43	42	35–50	16s
	Primary Sjögren’s syndrome	NL	1	39	965	40–55	16s
	Pemphigus vulgaris	CN	1	18	14	44–45	16s
	Anti-NMDAR encephalitis	CN	1	30	12	29–32	16s
	Autoimmune hepatitis	CN	1	119	132	19–75	16s
Cancer	Colorectal cancer and adenoma	AT, CA, CN, DK, FI, FR, IR, IT, JP, MA, ES, US	27	1630	2131	40–70	16s (22) and shotgun (5)
	Gastric cancer and adenocarcinoma	CN, KR, MN	6	498	467	50–58	16s
	Pancreatic cancer and ductal adenocarcinoma	CN, US	3	76	58	55–68	16s
	Prostate cancer	US	2	26	24	63–66	16s (1) and shotgun (1)
	Cervical cancer	CN, US	2	50	51	48–60	16s
	Lung cancer	CN	1	30	30	50–61	16s
	Thyroid papillary cancer	CN	1	34	40	46–48	16s
	Breast cancer	CN	1	43	90	49–51	Shotgun
	Hepatocellular carcinoma (liver cancer)	CN	1	75	75	48–49	16s
	GIT neoplasia	FI	1	63	13	43–70	16s
Total	-	27	84^b^	4208	5957	-	-

^a^Arthritis studies include ankylosing spondylitis (*n* = 1), enthesitis-related arthritis (*n* = 2), juvenile idiopathic arthritis (*n* = 1), rheumatoid arthritis (*n* = 5), and spondyloarthritis (*n* = 1). ^b^Two studies investigated two different diseases. Countries — *AT* Austria, *AU* Australia, *AZ* Azerbaijan, *BE* Belgium, *CA* Canada, *CN* China, *DE* Germany, *DK* Denmark, *ES* Spain, *FI* Finland, *FR* France, *IN* India, *IR* Iran, *IT* Italy, *JO* Jordan, *JP* Japan, *KR* Republic of Korea, *MA* Morocco, *MN* Mongolia, *MX* Mexico, *NG* Nigeria, *NL* Netherlands, *PL* Poland, *SD* Sudan, *SE* Sweden, *UK* United Kingdom, *USA* United States of America

### Significant associations of alpha diversity in cancer and autoimmune studies have opposite trends

**Fig. 3:**
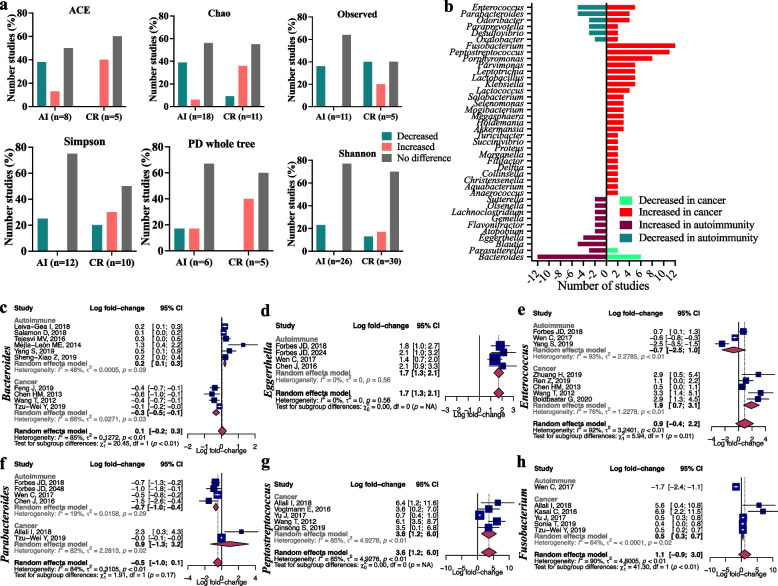
Alpha diversity and genus-level association of microbiome in cancer and autoimmune diseases. **a** Trend of alpha diversity of microbiome in cancer and autoimmunity across studies. “Decrease” and “increase” indicate statistical significance (*p* < 0.05) difference of alpha diversity between cases and controls, and “no difference” indicates no statistical significance (*p* > 0.05) difference of alpha diversity between cases and controls. Here, AI denotes Autoimmunity and CR Cancer. **b** Bacterial genera found to be significantly (*p* < 0.05) increased or decreased in cancer or autoimmune patients in more than one and in at least 70% of the studies in respective disease categories. **c**–**h** Forest plot of log fold change of relative abundance of six genera in cancer or autoimmune patients compared with healthy controls

The majority of studies (65/82) reported alpha diversity as a measure of microbiome diversity within cases or controls (Fig. 3a). The most frequently used alpha-diversity indices were the Shannon, Chao, and Simpson, reported in 56, 29, and 22 studies, respectively. Overall, most indices showed no significant difference in alpha diversities between cases and controls in both cancer (62%) and autoimmune (67%) studies. However, an increasing trend of alpha diversity in cancer studies and an opposite decreasing trend in autoimmune studies were observed. The overall alpha diversity was significantly increased among cases in 25% of the cancer studies and decreased in 13%. Conversely, the alpha diversity was significantly decreased among cases in 30% of the autoimmune studies and increased in 3% (Fig. 3a).

### Cancer and autoimmunity contain inverted and distinct microbiome signatures

A microbiome feature can be repeatedly found to be increased in cancer and decreased in autoimmunity or vice versa in multiple studies. We identified features that fall into two categories: (i) cancer increased and (ii) autoimmune increased. The “cancer-increased” category represents the microbiome features that were found to be increased among the cancer cases but decreased or not significantly associated among the autoimmune cases. On the other hand, the “autoimmune-increased” category represents the microbiome features that were found to be increased in autoimmune cases but decreased or not significantly associated in the cancer cases.

#### Genus

We identified 214 distinct genus-level associations with either cancer or autoimmune diseases across 73/82 (89%) studies. Of these, 83 genera were reported in more than one cancer or autoimmune study. However, we filtered out 43 of 83 genera (Supplementary Table 5) based on the second filtering threshold (present in at least 70% of the disease category). Therefore, we could identify 40 genera with a consistent trend to be increased or oppositely decreased in cancer and autoimmunity (Fig. 3b). Of these 40 genera, 30 was found in the cancer-increased category and 10 in the autoimmune-increased category (Fig. 3b). In the cancer-increased category, six genera (*Enterococcus*, *Parabacteroides*, *Odoribacter*, *Paraprevotella*, *Desulfovibrio*, and *Oxalobacter*) show true opposite directionality (i.e., increased in cancer but decreased in autoimmune diseases) between cancer and autoimmune diseases, whereas 24 genera in the cancer-increased category were increased in cancer but not significantly associated between the cases and controls in autoimmunity. Of them, *Fusobacterium*, *Peptostreptococcus*, and *Porphyromonas* were the most frequently increased genera among the cancer cases which were found to be associated in 13, 11, and eight cancer studies, respectively. In the autoimmune-increased category, *Bacteroides* and *Parasutterella* were inversely associated between cancer and autoimmune cases. The opposite directionality of *Bacteroides* between cancer and autoimmune diseases is the most prominent, which was found to be increased in 12 autoimmune studies and decreased in six cancer studies. The remaining eight genera in the autoimmune-increased category were always increased in autoimmunity but absent in cancer.

We next explored the quantitative level of the relative abundance difference of the genus between cases and healthy controls. We extracted the relative abundance data of these significantly associated genera, where available, and conducted a random-effects meta-analysis. We conducted the meta-analysis only on genera for which we found the relative abundance data in at least four studies. By this criteria, six genera had sufficient abundance data available in at least four studies. In the autoimmune-increased genus category, meta-analysis was performed on *Bacteroides* and *Eggerthella*. Meta-analysis of *Bacteroides* involved 10 studies including six autoimmune and four cancer studies. The genus *Bacteroides* was increased among the autoimmune cases by an overall +0.4 log fold change (95% *CI*, 0.1 to 0.3) compared with healthy controls, whereas it was decreased among the cancer cases by an overall −0.3 log fold change (95% *CI*, −0.5 to −0.1) (Fig. 3c). Between-study heterogeneity among the autoimmune and cancer studies were 48% and 66%, respectively. Four autoimmune studies were included in the meta-analysis of *Eggerthella*, which showed an increased abundance in autoimmune cases by an overall +1.7 log fold change (95% *CI*, 1.3 to 2.1) with a between-study heterogeneity of 0% (Fig. 3d). In the cancer-increased genus category, meta-analysis was performed on *Enterococcus*, *Parabacteroides*, *Peptostreptococcus*, and *Fusobacterium*. A total of eight studies were included in the meta-analysis of *Enterococcus*, which showed an overall increase of +1.9 log fold (95% *CI*, 0.7 to 3.1) among the cancer cases and decrease by −0.7 log fold (95% *CI*, −2.5 to 1.0) (Fig. 3e). The between-study heterogeneity was 76% and 93% among the cancer and autoimmune studies, respectively. We included six studies in the meta-analysis of *Parabacteroides* (Fig. 3f). It accounted for an overall +0.9 log fold increase (95% *CI*, −1.3 to 3.2) in cancer cases and −0.7 log fold decrease (95% *CI*, −1.0 to −0.48) in autoimmune cases with 82% and 19% between-study heterogeneity, respectively. Meta-analysis of *Peptostreptococcus* includes five cancer studies which show an overall +3.6 log fold increase (95% *CI*, 1.2 to 6.0) among the cancer cases where the between-study heterogeneity was 85% (Fig. 3g). Five cancer studies were included in the meta-analysis of *Fusobacterium* (Fig. 3h). The results showed an overall +0.5 log fold increase (95% *CI*, 0.3 to 0.7) of *Fusobacterium* among the cancer cases with a between-study heterogeneity of 64%.

#### Species

**Fig. 4:**
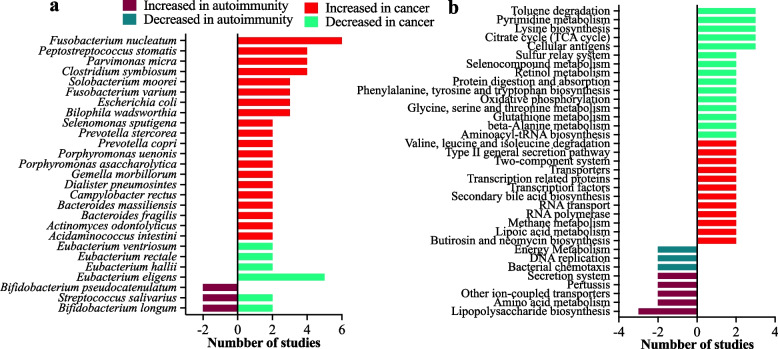
Species-level association and predicted metabolic pathways of microbiome in cancer and autoimmune diseases. Bacterial species (**a**) and predicted metabolic pathways (**b**) found to be significantly (*p* < 0.05) increased or decreased in cancer or autoimmune patients in multiple cancer and autoimmune studies

Twenty-eight studies (34%) reported significant species-level taxonomic associations in cancer and autoimmune diseases. A total of 159 species were significantly associated in at least one study, of which 28 species were associated in more than one cancer or autoimmune study. Of these 28 species, 27 were found to be associated with cancer or autoimmune disease categories; however, only one species (*Faecalibacterium prausnitzii*) was consistently found to be decreased both in cancer (*n* = 2) and autoimmune studies (*n* = 3). Among the 27 species, 20 were identified in the cancer-increased category and seven in the autoimmune-increased category (Fig. 4a). *Fusobacterium nucleatum* was the most common species in the cancer-increased category, appearing in eight cancer studies. However, seven of these eight cancer studies were on colorectal cancer, and one was on breast cancer. This might actually represent a skew, because colorectal cancer was the most represented cancer among the cancer studies (27/45). Therefore, it is reasonable that *F. nucleatum* would be the most common cancer-increased species, and it may not indicate that *F. nucleatum* was increased in cancer generally. None of the cancer-increased species was found to be significantly associated in the autoimmune studies. Among the autoimmune-increased species, *Bifidobacterium longum* and *Streptococcus salivarius* were associated inversely between cancer and autoimmune diseases. Interestingly, four members of the *Eubacterium* genus, *Eubacterium eligens*, *Eubacterium hallii*, *Eubacterium rectale*, and *Eubacterium ventriosum*, were decreased in cancer cases in multiple studies. However, none of these *Eubacterium* species was significantly associated with autoimmune diseases.

### Metabolic pathways

Associations between predicted microbial metabolic pathways and diseases were only reported in 20 studies (24%). A total of 405 predicted metabolic pathways were shown to be significantly associated in at least one cancer or autoimmune study. Most of the pathways were found in only one study, whereas 48 were associated with cancer and autoimmunity in multiple studies. Of these 48 pathways, 35 were increasingly or decreasingly associated with cancer and autoimmunity (Fig. 4b), whereas 13 were found to be inconsistently or ambiguously associated with disease categories (Supplementary Table 6). None of the pathways exhibited an inverse association between cancer and autoimmunity, as observed in several genera and species. However, some pathways were increased or decreased in multiple cancer and autoimmune studies such as lipopolysaccharide biosynthesis, which was found to be increased in three autoimmune studies (Fig. 4b).

### Disease-associated genera and species are taxonomically and functionally diverse

#### Taxonomic diversity

**Fig. 5:**
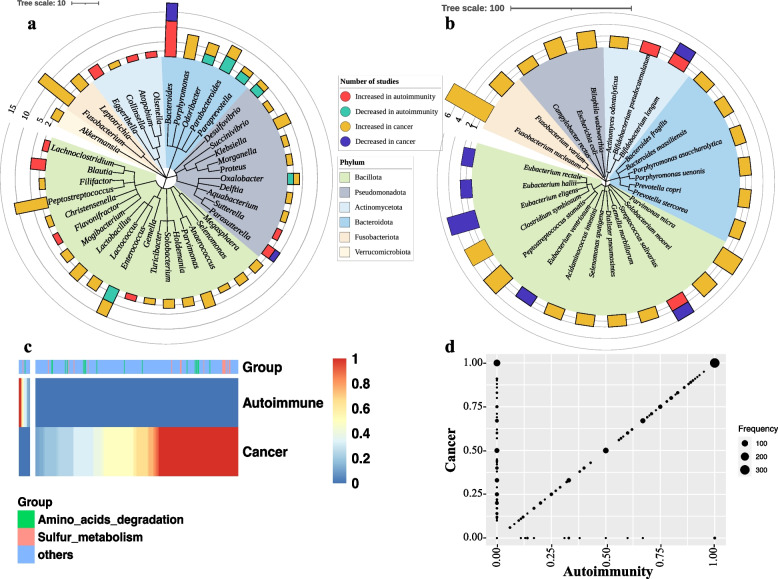
Taxonomic and functional diversity of cancer and autoimmune-associated microbiome. Taxonomic relatedness of bacterial genus (**a**) and species (**b**) in cancer and autoimmune diseases. The bars represent the total number of studies where the respective taxa were found to be significantly increased or decreased. The colors of the clades denote Phylum. The heatmap (**c**) shows 320 distinct metabolic pathways that were exclusively found in either cancer or autoimmune-increased taxa. The pathways were predicted from a list of cancer and autoimmune microbiomes by querying the metaCyc database using MACADAM [115]. The color scale shows pathway completeness score. Pathway completeness score 1 indicates the respective bacterial taxa contains a full set of metabolic potentials to complete the pathway. The left cluster is likely autoimmune-specific microbial pathways, and the right big cluster is likely cancer-specific microbial pathways. A complete list of these pathways can be found in the Supplementary file 2. The scatterplot (**d**) shows the number of pathways (dot size) with specific pathway score features between cancer and autoimmune microbes

To understand the phylogenetic and taxonomic relatedness among the associated genera and species, we used the NCBI common tree taxonomy browser tool for the construction of taxonomic trees. All 40 cancer-increased and autoimmune-increased genera belonged to six bacterial phyla. The majority of the genera (*n* = 18) belonged to the phylum Bacillota followed by Pseudomonadota (*n* = 10) (Fig. 5a). All 27 species were represented by five phyla (Fig. 5b). The Bacillota phylum represents the majority of the species (*n* = 13) followed by Bacteroidota (*n* = 6). We observed some interesting trends across phyla. The members (both genus and species) of Fusobacteriota were always found to be increased in cancers. Interestingly, most of the genera and species of the phylum Bacteroidota were found to be increasingly associated with cancer and decreasingly associated with autoimmunity. The only exception is the *Bacteroides* genus which showed an opposite directionality compared with other members of this phylum (Fig. 5b). Similarly, the majority of the members of the Pseudomonadota showed a positive association with cancer or a negative association with autoimmunity, with the exception of the genera *Parasutterella* and *Sutterella*, which showed an opposite directionality. Most of the genera of the Bacillota phylum was found to be increased both in cancer and autoimmunity. However, the species of the Bacillota phylum could be divided into two groups considering opposite directionality with cancer and autoimmunity. One group of species was found to be increased in cancer (*n* = 8 species), and another group was found to be decreased in cancer (*n* = 5 species) (Fig. 5b).

#### Functional diversity

To predict the shared or unique metabolic functions among the cancer-increased or autoimmune-increased genera and species, we identified metabolic pathways using MetAboliC pAthways DAtabase for Microbial taxonomic groups (MACADAM) [115], which employs pathway tools based on the MetaCyc database [116] that includes metabolic pathways as well as associated metabolites, reactions, and enzymes. We first extracted all the metabolic pathways that are present among cancer-increased and autoimmune-increased genera and species. We then identified the pathways that were exclusively present either in cancer-increased or autoimmune-increased groups. Of the 936 metabolic pathways that were detected together in two groups, 303 pathways were found exclusively in cancer-increased taxa. On the other hand, only 17 pathways were present exclusively in autoimmune-increased taxa (Fig. 5c, Supplementary file 2). Among the 303 cancer-increased pathways, 119 had a complete pathway score of 1, meaning that some members of the cancer-increased taxa contain all enzymes to complete the metabolic pathway. The most frequent pathways in the cancer-increased groups belonged to aromatic compound degradation (38/303) followed by non-carbon nutrient metabolism (23/303), amino-acid degradation (21/303), and cofactor biosynthesis (16/303). Interestingly, 17 of the 23 non-carbon nutrient metabolism pathways were related to sulfur metabolism. Of the autoimmune-increased pathways (*n* = 17), four complete metabolic pathways were contained in some members of the autoimmune-increased taxa (Fig. 5d, Supplementary file 2).

Because we observed taxonomically opposing directionality within the members of Bacteroidota, Pseudomonadota, and Bacillota (Fig. 5a–b), we further explored the functional similarities or dissimilarities within the members of these phyla. In the Bacteroidota phylum, four genera and six species were associated in the cancer-increased group (Fig. 5a–b), whereas only the *Bacteroides* genus belonged to the autoimmune-increased group. By comparing the potential metabolic functions between the cancer-increased taxa and the *Bacteroides* genus (autoimmune increased) under the Bacteroidota phylum, we detected 53 pathways exclusively present among the cancer-increased taxa and 39 pathways that were exclusively present in the genus *Bacteroides* (Supplementary file 2). In the Bacillota phylum, we compared the metabolic functions between cancer-increased (i.e., *Anaerococcus*, *Christensenella*, *Dialister pneumosintes*, *Enterococcus*, *Filifactor*, *Gemella morbillorum*, *Lactobacillus*, *Lactococcus*, *Megasphaera*, *Mogibacterium*, *Parvimonas*, *Parvimonas micra*, *Selenomonas*, *Selenomonas sputigena*, *Acidaminococcus intestini* and *Turicibacter*) and autoimmune-increased (i.e., *Blautia*, *Eubacterium eligens*, *Eubacterium hallii*, *Eubacterium rectale*, *Flavonifractor*, *Gemella*, *Lachnoclostridium*, and *Streptococcus salivarius*) species. A total of 162 pathways were exclusively present in the cancer-increased group, and 21 pathways were found exclusively in the autoimmune-increased group under the phylum Bacillota (Supplementary file 2). Similarly, in the Pseudomonadota phylum, we compared the metabolic functions in the cancer-increased group (i.e., *Aquabacterium*, *Bilophila wadsworthia*, *Delftia*, *Desulfovibrio*, *Escherichia coli*, *Klebsiella*, *Morganella*, *Oxalobacter*, and *Proteus*) and autoimmune-increased group (i.e., *Sutterella*). A total of 700 pathways were exclusively present in the cancer-increased group, and no pathways were found exclusively in the autoimmune-increased taxa in this phylum (Supplementary file 2).

## Discussion

This work involved a systematic literature review on gut microbiome studies in cancer and autoimmune conditions. We also conducted a meta-analysis to pool the relative fold change of certain genera across multiple studies. We identified a set of microbiome features that show a consistently opposite directionality between cancer and autoimmune diseases in multiple studies. This study also identified many non-robust microbiome associations that were only reported in a single study, illustrating the degree of variation between gut microbiome studies and the need for larger sampling or rigorous meta-analysis to distinguish significant data from noise. Although only a minority of features were found to be consistently opposite, but this is still much more than would be expected because finding a feature that shows repeated directionality in multiple studies might represent a biological connection with the disease. Most cancer studies show an increase of a group of well-known cancer-associated microbiome features and decrease of commensal bacterial genera in cancer cases compared with healthy controls, whereas the autoimmune cases are characterized by invasion or depletion of commonly known commensal bacterial genera. We identified 30 genera that were categorized as cancer increased. Of them, 24 were always increased in cancer but had no significant association with autoimmune diseases. Most of these 24 genera are previously reported cancer-associated bacteria such as *Fusobacterium*, *Peptostreptococcus*, and *Porphyromonas*. The genus-level association is also consistent for some species under the same genera. Most of the 20 species found to be positively associated with cancer are cancer-associated bacteria such as *Fusobacterium nucleatum* [117], *Peptostreptococcus stomatis* [118], and *Parvimonas micra* [119]. These findings corroborate a previous meta-analysis of gut microbiome studies, which found increased abundance of similar microbiome features in multiple colorectal cancers studies [120]. However, six of the 30 cancer-increased genera, namely *Enterococcus*, *Parabacteroides*, *Odoribacter*, *Paraprevotella*, *Desulfovibrio*, and *Oxalobacter* show true opposite directionality between cancer and autoimmune diseases. Several species under these genera are known to be associated as potent immune suppressors. For instance, a well-known opportunistic pathogen *Enterococcus faecalis* is capable of modulating immune function by suppressing macrophage activity through preventing NF-κB signaling [121] or promoting anti-inflammatory cytokine IL-10 [122, 123]*.* Similarly, members of the genus *Parabacteroides* including *Parabacteroides distasonis* were previously found to be decreased in autoimmune disease [124] and to reduce the severity of intestinal inflammation in colitis in mice through decreased production of proinflammatory cytokine TNF-α [125].

A set of ten genera and seven species were identified in the autoimmune-increased category. *Bacteroides*, *Parasutterella*, *Bifidobacterium longum*, and *Streptococcus salivarius* were found to be inversely associated between cancer and autoimmune cases. In addition, multiple *Eubacterium* species were identified in this category (Fig. 4). *Bacteroides* was found to be increased in 12 autoimmune studies and decreased in 6 cancer studies, whereas *Parasutterella* was found to be increased in 3 autoimmune studies and decreased in 2 cancer studies. Several species of *Bacteroides* and *Parasutterella* were previously shown to be capable of restoring antitumor responses such as *Bacteroides thetaiotaomicron*, *Bacteroides rodentium*, and *Parasutterella excrementihominis* [126–128].

Comparison of metabolic pathways across cancer-increased and autoimmune-increased microbiome taxa shows unique microbiome functions. Most noticeably, sulfur-metabolism pathways were found to be one of the frequent pathways among certain member of cancer-increased taxa such as *Aquabacterium*, *Bilophila wadsworthia*, *Delftia*, *Desulfovibrio*, *Escherichia coli*, *Fusobacterium*, *Klebsiella*, *Lactobacillus*, *Morganella*, *Odoribacter*, and *Proteus*. The presence of sulfur-metabolizing microbiome in the human gut has been shown to be associated with a high risk of colorectal cancer [129]. Although the exact mechanism is unknown, the intermediary sulfur metabolites and the production of hydrogen sulfide (H2S) are thought to be associated with colorectal cancer pathogenesis and epithelial damage [130, 131]. However, disease-specific causality and the underlying mechanism of these associations are yet to be discovered. Therefore, further laboratory experimentation with a combination of multiple members of these sets of microbiome features will help to understand their biological role in immunomodulation in autoimmunity and cancer.

This is the first systematic review that synthesized results from 82 case-control microbiome studies in cancer and autoimmunity, representing participants from multiple age groups and geographies. We implemented a broad and advanced search strategy in multiple databases which enable us to identify the majority of the publications in the field. We defined clear inclusion and exclusion criteria. We conducted quality assessment of the included studies using the Cochrane recommended standard tools and maintained high standards by the strict implementation of PRISMA guidelines.

The study has some limitations. We conducted a traditional systematic review and meta-analysis rather than reanalysis of previously published sequencing data. Therefore, we mainly relied on the primary studies’ sequence analysis, taxonomic classification, and statistical test. Studies typically use different models and diverse types of bioinformatics pipelines, taxonomic classifiers, and statistical comparisons which may infer dissimilar taxonomies and sometimes render results difficult to compare. Reanalysis of raw sequencing data is the standard approach of performing meta-analysis of microbiome studies that provide quantitative comparison of taxonomies across diseases. However, there are also many challenges of a meta-analysis of raw sequencing data such as diverse study design, heterogeneous demographics of study participants, different methods for DNA extraction and sequencing type and platform, and availability of metadata and raw sequences. For instance, in our study, many of the included studies (> 50%) do not have publicly available raw sequencing data. Moreover, the findings of this study are very similar to a previous meta-analysis [120] where the authors performed reanalysis of raw sequencing data of 28 published case-control studies across ten diseases. They detected an increased abundance of common cancer-associated bacteria such as *Fusobacterium*, *Porphyromonas*, *Peptostreptococcus*, and *Parvimonas* in multiple colorectal cancer studies. We found similar associations for all of these bacteria in multiple cancer studies in our analysis. Furthermore, we conducted a meta-analysis on the relative abundance of six genera to explore the quantitative difference in their abundance between disease cases and controls. The diverse methodological approaches used in the primary microbiome studies, such as DNA processing and bioinformatic pipelines, might affect the pooled estimate of the meta-analysis. Therefore, it would be appropriate to perform a subgroup analysis to identify the source of heterogeneity if it existed. Unfortunately, it was not feasible to perform a subgroup meta-analysis considering the few numbers of studies in each subgroup. For example, we included six studies for meta-analysis on *Bacteroides* abundance in autoimmune diseases (Fig. 3c). Of these six studies, two were in each category of v3–v4, v2–v4, and v4–v5 based on the region of 16s gene amplified. However, we collected this additional information and incorporated it into Supplementary file 1. Finally, we were not able to conduct a meta-analysis on many genera and species due to the absence of relative abundance data in the published literature.

Based on our findings and observations, we propose some recommendations and future directions in this field of research. We recommend that future case-control microbiome studies should report relative abundance data, sample variance, and *p*-value of the statistical analysis which will facilitate future meta-analysis and enable combining of results from multiple studies. The importance of publicly available raw sequencing data and patient metadata is enormous for direct comparison of the results across studies. In this study, we can only draw correlative evidence with some microbiome features between cancers and autoimmunity but cannot speak to causality and mechanism, one way or the other. Therefore, the findings from this study can be used in future studies to understand the mechanistic connection which can help to identify selective microbial consortia for microbiome-based immune therapies for the prevention and treatment of cancer and autoimmune diseases which can be further tested by laboratory investigation.

In conclusion, despite some limitations, this study identified distinct types of cancer and autoimmune associated microbiome features that are consistently identified in multiple studies. These associations may point to important underlying biology of how disease and health shape the microbiome or vice versa, and this serves as an unprecedented trove of hypotheses for future studies of the role of the microbiome in autoimmunity and cancer.

## Supplementary Information

**Additional file 1: Supplementary File 1**. Characteristics of included studies.

**Additional file 2.** **Supplementary File 2.** List of predicted metabolic pathways that were retrived through MACADAM.

**Additional file 3: Supplementary Table 1**. Electronic database search strategy to identify published reports on cancer- and autoimmune-microbiome studies. **Supplementary Table 2.** PRISMA 2009 Checklist. **Supplementary Table 3**. Articles included in this study (*n*=82). **Supplementary Table 4**. Articles excluded from this study (*n*=148). **Supplementary Table 5**. List of ambiguous or inconsistent genera. **Supplementary Table 6**. . List of ambiguous or inconsistently associated pathways.

## Data Availability

All data that support the results of this study are available from the corresponding author on request. We included a complete list of all included and excluded studies as supplementary files.

## Abbreviations

EMBAS: Excerpta Medica dataBASE
PRISMA: Preferred Reporting Items for Systematic Reviews and Meta-Analyses
FDR: False discovery rate
GIT: Gastrointestinal tract
H2S: Hydrogen sulfide
MACADAM: MetAboliC pAthways DAtabase for Microbial taxonomic groups
MeSH: Medical Subject Headings
NMDAR: Anti-N-methyl-D-aspartate receptor
NOS: Newcastle-Ottawa Scale
T1D: Type 1 diabetes
