# Supplementary material for: Reproducible and opposing gut microbiome signatures distinguish autoimmune diseases and cancers: a systematic review and meta-analysis
Source: Microbiome. 2022 Dec 9;10:218. doi: 10.1186/s40168-022-01373-1 (PMC9733034; doi:10.1186/s40168-022-01373-1)
Supplement: Supplementary file 4 — Additional file 3: Supplementary Table 1. Electronic database search strategy to identify published reports on cancer- and autoimmune-microbiome studies. Supplementary Table 2. PRISMA 2009 Checklist. Supplementary Table 3. Articles included in this study (n=82). Supplementary Table 4. Articles excluded from this study (n=148). Supplementary Table 5. List of ambiguous or inconsistent genera. Supplementary Table 6. . List of ambiguous or inconsistently associated pathways. [file 40168_2022_1373_MOESM3_ESM.zip › Supplementary_Table6_ESM.docx]

**Supplementary Table 6.** List of ambiguous or inconsistently associated pathways.

| Pathways (n=13) | No. cancer decrese study | No. Cancer increase study | No. autoimmune decrease study | No. autoimmune increase study |
| --- | --- | --- | --- | --- |
| Cysteine and methionine metabolism | 2 | 1 | 0 | 0 |
| D-alanine metabolism | 2 | 1 | 0 | 0 |
| Peptidoglycan biosynthesis | 2 | 1 | 0 | 0 |
| Purine metabolism | 2 | 1 | 0 | 0 |
| Type II diabetes mellitus | 2 | 1 | 0 | 0 |
| Arachidonic acid metabolism | 2 | 1 | 0 | 1 |
| Flagellar assembly | 1 | 2 | 1 | 0 |
| Galactose metabolism | 1 | 2 | 0 | 0 |
| Riboflavin metabolism | 1 | 2 | 0 | 0 |
| Starch and sucrose metabolism | 1 | 2 | 0 | 0 |
| Ubiquinone and other terpenoid-quinone biosynthesis | 1 | 2 | 0 | 1 |
| Cellular Processes & Signaling | 0 | 0 | 2 | 1 |
| Inorganic ion transport and metabolism | 0 | 0 | 1 | 2 |
